# Social prescribing link workers: a test for public health ethics?

**DOI:** 10.1177/17579139251355898

**Published:** 2025-07-22

**Authors:** C Melam

**Affiliations:** Faculty of Health, Education and Life Sciences, Birmingham City University, Birmingham, UK

## Abstract

This article builds on the conversation initiated in The Unusual Suspects by the Royal Society for Public Health regarding the wider public health workforce. It focuses on the ethical implications of evolving “bridge” roles, such as Social Prescribing Link Workers (SPLWs), who operate at the intersection of clinical care and community-based support. The commentary explores how these roles are expanding globally, with the UK offering a key case study due to its scale of implementation and integration into formal health systems.

In a context marked by health inequalities, economic strain, and the long shadow of the COVID-19 pandemic, the need for ethical guidance that is responsive to real-world practice has never been greater. The boundaries of public health practice are being redrawn globally. As roles that once belonged solely to either clinical or public health domains become increasingly integrated, the ethical frameworks designed to support these professions are struggling to keep pace.

Among the clearest indicators of this shift are Social Prescribing Link Workers (SPLWs), a role formalised in England’s health system but increasingly paralleled in other countries through primary, community health and integrated care models. SPLWs straddle individual care and community health. Social prescribing plays an important role in addressing health inequalities, particularly by focusing on socio-psychological factors and the management of long-term conditions.^1^ SPLWs act as a bridge between medical and non-medical sectors,^2^ offering a relational, community-based response to complex social needs.

The World Health Organization (WHO) recognises social prescribing as a promising practice to address social determinants of health and improve population wellbeing. It defines social prescribing as a means of linking individuals with non-medical sources of support within communities and has developed toolkits to guide global implementation. While social prescribing is being explored or piloted in over 30 countries, the United Kingdom remains the first to embed it at scale within its healthcare system, making England a key case study for both operational learning and ethical reflection.^3^

**Figure:**
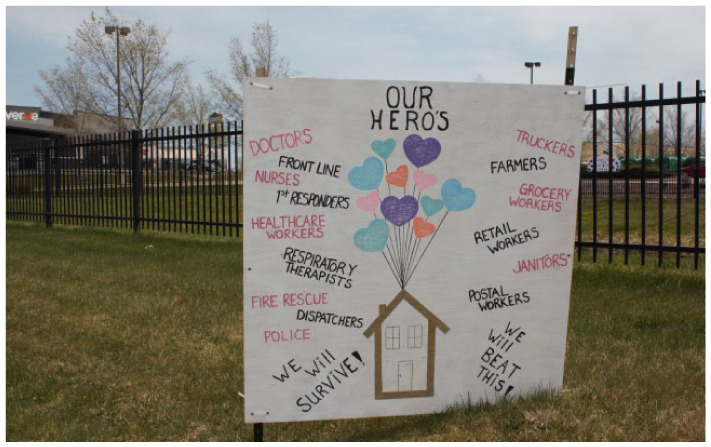


Although community connector-type roles have existed in various countries, England’s formal adoption of SPLWs into primary care settings illustrates a shift with ethical implications. The NHS Long Term Workforce Plan has projected the recruitment of up to 9000 SPLWs.^4^ These workers themselves are now calling for formal professional recognition, including accreditation and standardised training, to support their evolving responsibilities.^5^

This integration into clinical environments introduces new ethical demands, particularly around accountability, proximity to clinical decisions, and power dynamics. These are areas that current public health frameworks are ill-prepared to address. Yet despite their growing significance, the ethical dimensions of SPLW practice remain largely unsupported.

SPLWs routinely face moral dilemmas that extend beyond the reach of conventional ethical models. They must navigate tensions between personal autonomy and systemic pressures, respond to structural injustices, and influence behaviour through trust-based relationships that sit outside regulated clinical frameworks. Unlike registered clinicians, SPLWs are not governed by professional codes or statutory standards. Their ethical terrain is informal and often invisible. Yet the stakes involved in their work, including safeguarding, informed consent, confidentiality, and equity, mirror those of more formally recognised health roles.

Bridge and evolving roles such as SPLWs are no longer peripheral in public health. A UK report, *The Unusual Suspects*,^6^ highlights how many non-traditional professionals, including housing officers, community connectors, and youth workers, carry out essential public health work without the recognition or ethical infrastructure typically afforded to healthcare professionals. Although the context is UK-specific, similar patterns are observable internationally in community health systems, particularly where multidisciplinary teams address social determinants of health.

Globally, health systems are seeing comparable role evolution in efforts to address population health more holistically. Yet the ethics frameworks have not kept pace. Clinical bioethics remains focused on individual autonomy, while public health ethics often address abstract population-level issues like vaccine mandates. Neither adequately supports the granular, context-sensitive decisions made by SPLWs and similar professionals.

The relational dynamics of bridge roles like SPLWs also raise important ethical questions. Trust-based relationships are often at the heart of their work, but these come with blurred boundaries and asymmetries of power, especially in the absence of formal regulation. There may be value in exploring whether a more applied, reflexive, and justice-oriented ethical framework could support practitioners in navigating these complexities while respecting the lived experiences of both professionals and the communities they serve.

In this light, SPLWs might be viewed not just as a workforce innovation but as a touchstone for the ethical preparedness of public health systems. If these roles are to succeed and be sustained, the question becomes whether current ethical thinking is fit for purpose or if it, too, must evolve. As SPLWs and similar roles continue to emerge globally, they offer a timely and valuable lens through which to reflect on what a future-facing public health ethics might look like.
